# Pediatric patient with systemic lupus erythematosus & congenital acquired immunodeficiency syndrome: An unusual case and a review of the literature

**DOI:** 10.1186/1546-0096-6-7

**Published:** 2008-05-01

**Authors:** Elizabeth C Chalom, Fariba Rezaee, Joel Mendelson

**Affiliations:** 1 Department of Pediatrics, Saint Barnabas Medical Center, Livingston, NJ USA; 2 Department of Pediatrics, West Virginia University, Morgantown, WV, USA; 3 Department of Pediatrics, Newark Beth Israel Medical Center, Newark, NJ, USA

## Abstract

The coexistence of systemic lupus erythematosus (SLE) in patients with congenital human immunodeficiency virus (HIV) infection is rare. This is a case report of a child diagnosed with SLE at nine years of age. She initially did well on non-steroidal anti-inflammatory agents, hydroxychloroquine, and steroids. She then discontinued her anti-lupus medications and was lost to follow-up. At 13 years of age, her lupus symptoms had resolved and she presented with intermittent fevers, cachexia, myalgias, arthralgias, and respiratory symptoms. Through subsequent investigations, the patient was ultimately diagnosed with congenitally acquired immunodeficiency syndrome (AIDS).

## Introduction

The coexistence of HIV in patients with lupus is extremely rare, especially in the pediatric population. Case reports generally describe patients with HIV who subsequently develop rheumatologic complaints. In children, there have only been five reported pediatric cases of SLE and HIV [1-4]. In all of five, the children had known congenital HIV and then developed SLE. In four of those cases, the child developed lupus nephritis, but no other clinical manifestation of SLE [1-3]. The fifth was a case report of lupus vasculitis without nephritis in a child with congenital HIV [4]. The case we are presenting is exceedingly rare among pediatric cases and we could not find a similar one in the literature. Our patient first presented with SLE and subsequently showed the manifestations of congenital human immunodeficiency syndrome.

## Case presentation

Patient was a 9 year old African American female who presented with fevers, polyarthritis, and morning stiffness. She had no rash or significant adenopathy. Her height was at the 25^th ^percentile for age and her weight was just below the 50^th ^percentile. Laboratory investigations revealed WBC = 2,800 cells/mm^3 ^(58% neutrophils, 38% lymphocytes), hemoglobin = 10.4 g/dL, platelets = 167,000/mm^3^, erythrocyte sedimentation rate(ESR) = 56 mm/h, ANA titer = 1:1280, C4 complement <10 mg/dl, and anti-double stranded DNA (dsDNA) = 1051 IU/ml. Urinalysis was within normal limits. She was diagnosed with SLE and treated with prednisone, naproxen, and hydroxycholoroquine Over the next ten months, the prednisone was tapered and discontinued. The naproxen was later changed to rofecoxib and she did well on a combination rofecoxib and hydroxychloroquine for two years. She was then lost to follow-up for 13 months and did not take her medications. She then returned, complaining of arthralgias. She had no arthritis on exam and laboratory studies showed anemia with a hemoglobin of 9.9, WBC = 2.1 cells/mm^3 ^(51%neutrophils, 23% lymphocytes, 21% monocytes and 5% basophils) and ESR = 56 mm/h. Her dsDNA was now negative and complements were normal.

She developed a fungal pharyngitis which resolved with fluconazole. She was referred to an immunologist, but never went. She was then brought into her pediatrician's office complaining of a nonproductive cough for three months. She described shortness of breath, left pleuritic chest pain, fevers to 101°F, chills, and night sweats for one week. There was a documented five-pound unintentional weight loss. She denied any joint pain, rashes, palpitations, diaphoresis, change in bowel patterns, or urinary symptoms. She also denied any history of sick contacts, blood transfusions, recent travel, sexual activity, or abuse. She was admitted to the hospital for further investigations.

**Past medical history **revealed that patient was born full-term at 5 pounds, 9 ounces to a 25 year old mother who smoked throughout pregnancy. She was discharged from the nursery without complications. There were no other past medical issues.

**Family history**, given by the maternal grandmother, was notable for her mother reportedly dying suddenly, three months prior to admission due to a "heart attack." Her father reportedly died several years prior from a motor vehicle accident. There was no family history of any rheumatologic disorders. Further history later obtained from grandfather, revealed that both biological parents had actually died of complications of HIV, and the mother was diagnosed with HIV when the patient was four year old. She was not checked for HIV during pregnancy.

### Social History

The patient lived in an inner city apartment with a brother, two sisters and maternal grandmother. She denied any use of illicit substances, alcohol, or tobacco.

**Physical examination **revealed an African-American female who was cachectic and pale, but alert, and in no acute distress. Vital signs showed a temperature of 99.2F, respiratory rate = 21 breaths per minute, saturating 99% on room air. Heart rate was 114 beats per minute and blood pressure 86/63 mmHg. Weight was at the 5^th ^percentile for age and height was 50^th ^– 75^th ^percentile. Significant findings included temporal muscle wasting and scarred tympanic membranes. She had enlarged (2–3 cm), non-tender nodes in the upper cervical, axillary, and inguinal regions. Respiratory exam revealed a productive cough and fair air entry with scattered rales, and left costochondral tenderness. Abdomen was benign with no organomegaly. Genital exam revealed Tanner stage III breasts and Tanner stage II pubic hair. She had no rashes, joint swelling, or limitation of movement.

**Laboratory studies **revealed: WBC = 9700 cells/mm^3^, hemoglobin = 9.6 g/dl, MCV = 75, RDW = 22.6, reticulocytes = 0.8%, and ESR = 191 mm/hr. HIV ELISA was repeatedly positive; HIV Western Blot positive, Viral Load = 516,527, CD4/CD8 = 0.06, CD4 Helper = 1.9, CD8 = 30.1. Her CH50 = 104 mg/dl, C3 = 178 mg/dl, C4 = 29 mgldl, dsDNA negative. Ear canal culture: heavy growth of Staph aureus. Blood culture was negative. Anergy panel was non-reactive.

Chest radiograph revealed extensive left lower lobe infiltrate. Chest CT Scan showed left lower lobe consolidation with no pleural effusion. Bronchoscopy revealed mild-moderate edema with moderate secretions in left bronchus.

The patient was treated with antibiotics, including Bactrim prophylaxis for pneumocystic carini. After one week she was discharged from hospital with the diagnoses of AIDS and Mycobacterium Avium Complex (MAC) infections. On follow up with the HIV clinic, she was placed on antiretroviral therapy and continued MAC medication. Her CD4 helper dropped to 0.40 × 10^3^. She died two years later from HIV cardiomyopathy.

## Discussion

The prevalence of lupus in the adult HIV population is low. From 1988–2002 only 30 cases were reported, and many did not meet four of the ACR criteria [5]. Most cases were patients with long-standing HIV infection who then presented with manifestations of SLE. There have been four reported pediatric cases in which children with congenital HIV, ages 7 to 37 months, developed lupus nephritis, but no other clinical manifestations of SLE [1-3]. There has been one pediatric case report of a 42 month old patient with AIDS and who later developed cutaneous vasculitis and met criteria for SLE [4]. This is the first reported case of a child with congenital HIV who first presented with multiple manifestations of lupus, and then developed AIDS years later.

It is not well understood why it is so rare to see both SLE and HIV in patients simultaneously. It has been suggested that demographic factors may be important since SLE is much more common in women, and HIV had been more common in homosexual men [6]. However, now that HIV is affecting more and more women, we would expect to see a rise in patients with both diseases, and that has not been reported. It has also been suggested that the marked antibody production in SLE is protective against HIV infection. This was first suggested by Wallace in 1991, when he noted the absence of HIV infection in lupus patients who received unscreened blood between 1978 and 1983 [7].

Hydroxychloroquine (HCQ) is often used to treat SLE and it may protect against HIV infection. HCQ has been used in combination with other antiviral medications in a few small studies and seems to help reduce the viral load in HIV. [8] Chloroquine and HCQ inhibit the posttranslational modification of gp120 in T cells and monocytes [9]. They increase endosomal pH and therefore inhibit pH dependent steps required for viral replication[10]. It is therefore possible that HCQ helped to slow down the AIDS manifestations in our patient. HCQ was started when she was diagnosed with SLE and discontinued 3 years later. Soon after she stopped taking the HCQ, her AIDS symptoms began.

On the other hand, there is increasing evidence that viruses may play a major role in the development of many rheumatologic diseases. Epstein-Barr virus, parvovirus, hepatitis, and retroviruses are potential triggers for various rheumatic disorders, including lupus[11]. In HIV, glycoprotein 120(gp 120) plays an important role in immune dysregulation. It can bind to CD4 on T cells to cause T cell activation and decreased self-tolerance. It can also bind to CD4 on B cells and cause polyclonal B cell activation [12]. It is possible that HIV infection might contribute to the development of SLE in patients such as ours.

AIDS and SLE are similar in many ways. They both can present with a combination of symptoms including fevers, rashes, weight loss, joint pain, anemia, leukopenia, proteinuria, vasculitis, etc. Both disorders can have significant T and B cell dysfunction with polyclonal B cell activation and T cell activation. Many patients thought to have lupus are screened for HIV for this reason. Both diseases can have a positive ANA, but in HIV the titer is generally low [13]. Anti-dsDNA antibodies are not usually seen in patients with HIV and are thought to be specific for lupus. Complements are also generally normal in HIV. Our patient had an ANA titer of 1:1280, an anti-dsDNA of 1051 and depressed C4 levels at initial presentation.

In summary, we present a 9-year-old female with SLE that was well controlled on hydroxychloroquine and rofecoxib. After being lost to follow-up and discontinuing her medications, she presented with fungal pharyngitis and then pneumonia and MAC infection. She tested positive for HIV and had a very low CD4 count. At that time she had no further complaints of arthritis and her lupus serologies had normalized. Immunosuppression secondary to HIV infection likely mitigated her manifestations of SLE.

We reviewed the literature and found only five other pediatric cases of congenital HIV and lupus. Our case is unique in that our patient had documented SLE and then developed manifestations of HIV. Therefore, in children with SLE and infections that seem unusual for the amount of immunosuppression due to the underlying rheumatologic disease or its treatment, the differential diagnosis must include congenital or acquired immunodeficiencies. Clinicians should not hesitate to test for HIV when appropriate, regardless of any known risk factors.

## Competing interests

The authors declare that they have no competing interests.

## Authors' contributions

FR drafted the initial case presentation and outlined a discussion

EC diagnosed and treated the patient for SLE. She edited the case presentation and added the discussion

JM made the diagnosis of HIV and followed the patient thereafter.

All authors read and approved the final manuscript.
